# A Evolução da Angioplastia Transluminal Coronariana na America Latina

**DOI:** 10.36660/abc.20200927

**Published:** 2021-05-06

**Authors:** Costantino Roberto Costantini, Rafael Michel de Macedo, Marcos Antônio Denk, Sergio Gustavo Tarbine, Lazaro Garcia, Mario Fernando Camargo Maranhão, Costantino Ortiz Costantini

**Affiliations:** 1 Hospital Cardiológico Costantini Ltda CuritibaPR Brasil Hospital Cardiológico Costantini Ltda, Curitiba, PR - Brasil; 2 Santa Casa de Curitiba CuritibaPR Brasil Santa Casa de Curitiba, Curitiba, PR - Brasil

**Keywords:** Angina Pectoris, Cineangiografia/história, Angioplastia Coronária com Balão/história, Angioplastia Coronária com Balão/tendências, Stents, Miocárdio/metabolismo, Evolução Clínica

Em agosto de 1979, dois anos e um mês após a realização da primeira angioplastia no mundo por Andreas Grüntzig (setembro de 1977),^1^ recebemos na Santa Casa de Misericórdia de Curitiba (SCMC) um paciente (A. S. O.) de 55 anos, sexo masculino, com quadro de angina aos esforços. O mesmo foi submetido a uma cineangiocoronariografia, que mostrou uma lesão crítica (estenose avaliada entre 75-80%), localizada no segmento proximal da artéria coronária direita (ACD). A artéria coronária esquerda (ACEsq) apresentava aspecto angiográfico normal e o ventrículo esquerdo (VE) uma hipocinesia discreta na parede inferior com a válvula mitral competente.

Surgia, então, a oportunidade de colocar em prática pela primeira vez na America Latina a técnica descrita por Grüntzig et al.^1^ Isso porque as lesões encontradas neste paciente preenchiam as características anatômicas necessárias: lesão única, curta (< 10 mm), proximal, com ausência de espasmo, concêntrica, não calcificada e indicação de cirurgia de revascularização do miocárdio. Dessa forma, após uma discussão entre a equipe clínica e o serviço de cirurgia cardiovascular, optou-se por propor ao paciente a dilatação da obstrução da ACD como tentativa de tratamento de sua insuficiência coronária.

**Figura 1:**
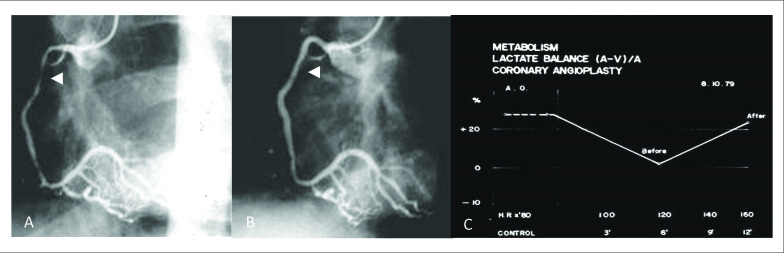
A) ACD Lesão em segmento proximal; B) Local da lesão pós-angioplastia; C) Dosagem de ácido láctico pré e pós-angioplastia (ano de 1979).

Após a sua concordância, em 10 de agosto de 1979 o paciente foi encaminhado para a sala de hemodinâmica, sendo submetido à intervenção coronária percutânea (ICP) descrita por Costantini et al.^2^ Após a ICP, a obstrução crítica da ACD foi reduzida a uma lesão de grau discreto (estenose avaliada entre 15-20%). Apesar do bom resultado angiográfico, existia a preocupação com o metabolismo do músculo cardíaco. Na ausência de outro método de avaliação isquêmica, foi realizada uma avaliação metabólica por meio da extração de uma amostra de sangue do seio coronário para avaliação do ácido lático, que confirmou a adequada oferta de oxigênio ao músculo cardíaco conforme demonstra a Figura 1.^2^

**Figura 2:**
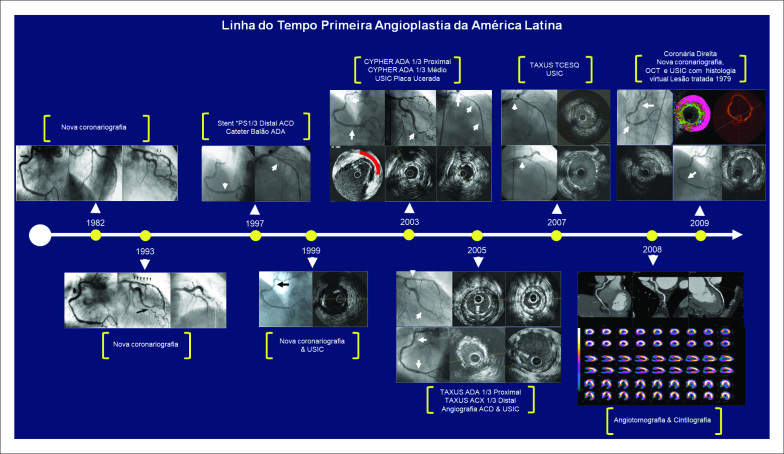
Evolução terapêutica entre 1982 e 2009 (A. S. O.).

Nos anos seguintes, o paciente (A. S. O.) foi rigorosamente monitorado quanto à evolução de sua doença coronariana. Outros tratamentos foram realizados ao longo do tempo, com a utilização de novas técnicas e tecnologias. A Figura 2 e a Tabela 1 apresentam toda a sua evolução terapêutica entre os anos de 1982 e 2009.

**Tabela 1:** Descritivo do quadro evolutivo e tratamento do paciente (A. S. O.) entre 1982 e 2009

Data	Exame	Diagnóstico	Conduta
1982	Cateterismo cardíaco: 3 anos de evolução	ACD com resultado angiográfico mantido; TCESQ: lesão de grau discreto; ADA e ACX: lesões de grau discreto.	Tratamento clínico
1993	Cateterismo cardíaco: 14 anos de evolução	VE função preservada; ACD: lesão de grau discreto no 1/3 proximal. TCESQ: lesão de grau discreto; ADA: lesão de grau moderado no 1/3 proximal e severo no 1/3 médio; ACX: lesão de grau severo no 1/3 distal.	ICP com aterectomia rotacional em ADA e com cateter-balão em ACX
1997	Cateterismo cardíaco: 18 anos de evolução	VE função preservada, ACD: lesão de grau discreto no 1/3 proximal e severo no 1/3 distal com presença de placa ulcerada pelo USIC. TCESQ: lesão de grau discreto; ADA: reestenose angiográfica de angioplastia prévia; ACX: resultado angiográfico. mantido em 1/3 distal.	ICP com implante de stent PS 4,0*15 mm em segmento distal de ACD e com cateter-balão em 1/3 médio proximal de ADA
1999	Cateterismo cardíaco: 20 anos de evolução (angina estável)	VE função preservada; ACD: lesão de grau discreto no 1/3 proximal avaliado com USIC e boa evolução em local submetido a stent PS. TCESQ: lesão de grau discreto; ADA: resultado angiográfico mantido; ACX: resultado angiográfico mantido em 1/3 distal.	Tratamento clínico
2003	Cateterismo cardíaco: 24 anos de evolução (angina estável)	VE Função preservada, ACD: lesão de grau discreto na 1/3 proximal e boa evolução em local submetido a stent PS. TCESQ: lesão de grau discreto; ADA: reestenose angiográfica de angioplastia prévia e placa ulcerada em óstio; ACX: reestenose angiográfica de grau moderado em 1/3 distal.	ICP com implante de dois stents Cypher® guiados com USIC em 1/3 proximal e médio de ADA (2,75*18 e 2,75*33 mm). Placa ulcerada do óstio avaliada com USIC e mantida em tratamento clínico por apresentar lúmen arterial preservado
2005	Cateterismo cardíaco: 26 anos de evolução (angina estável recorrente)	VE Função preservada; ACD: lesão de grau moderado na 1/3 proximal e boa evolução em local submetido a stents PS avaliados com USIC. TCESQ: lesão de grau moderado; ADA: lesão ostial com critérios de severidade pela avaliação com USIC; ACX: reestenose angiográfica de grau severo em 1/3 distal.	ICP com implante de um stent Taxus® em 1/3 distal da ACX (2,75*24 mm) e implante de stent Taxus® em óstio de ADA (3,0*32 mm) guiados com USIC
2007	Cateterismo cardíaco: 28 anos de evolução (angina estável classe II)	VE função preservada, ACD: lesão de grau moderado a importante no 1/3 proximal na avaliação angiográfica e limítrofe na avaliação com USIC e boa evolução em local submetido a stent PS. TCESQ: lesão de grau severo no 1/3 distal, ADA: boa evolução de stents prévios avaliados com USIC, ACX: boa evolução de stent prévio.	ICP com implante de um stent Taxus® em TCESQ (4,0*28 mm) guiado com USIC
2008	Check-up: angiotomografia/ cintilografia	Perfusão normal; VE função preservada; ACD: lesão de grau moderado a importante (60-70%) no 1/3 proximal e stent PS com boa evolução, porém com lesão severa (70%) em seu bordo proximal. TCESQ: stent prévio com boa evolução; ADA: stents prévios com boa evolução; ACX: stent prévio com boa evolução.	Tratamento clínico
2009	Cateterismo cardíaco: 30 anos de evolução (angina estável classe II)	ACD: Avaliação do 1/3 proximal com USIC na modalidade de histologia virtual e OCT evidenciando área luminal limítrofe com presença de grande core necrótico pela histologia e presença de uma fina camada da íntima pela avaliação com OCT; no 1/3 distal observa-se boa evolução do stent PS e placa severa em 1/3 médio com presença de úlcera.	ICP com implante de um stent metálico (3,5*18 mm) em 1/3 médio de ACD. Lesão de 1/3 proximal da ACD foi mantida em tratamento clínico

ACD: artéria coronária direita; TCESQ: tronco coronária esquerda; ACX: artéria circunflexa; ICP: intervenção coronária percutânea; USIC: ultrassom intracoronário; OCT: tomografia de coerência óptica; PS: Palmaz-Schatz.

**Figura 3:**
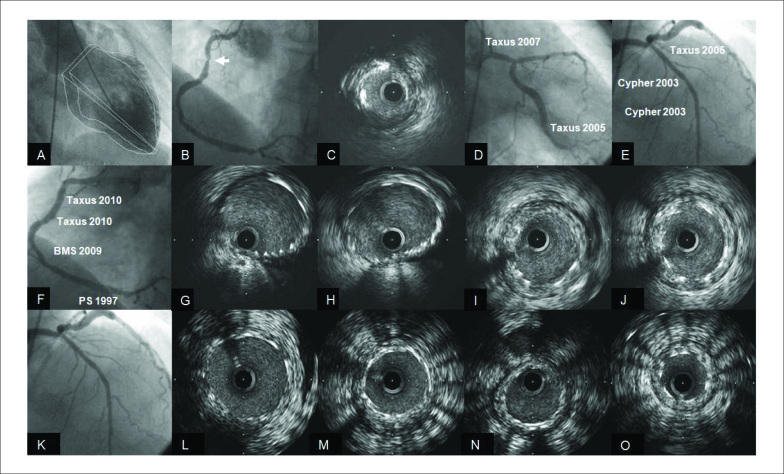
A) VE; B) ACD resultado angiográfico; C) ACD resultado USIC lesão severa 1/3 proximal; D) TCESQ/ACX resultado angiográfico; E) ADA resultado angiográfico; F) ACD pós-implante de stents 1/3 proximal; G, H) ACD USIC pós novos stents 1/3 proximal; I, J) ACD USIC stents prévios em 1/3 médio e distal; K-O) TCESQ/ADA resultado angiográfico e com USIC stents.

Em março de 2010, o paciente reingressa com novo quadro de angina estável (AE) classe II, sendo submetido ao nono cateterismo cardíaco, como apresentado na Figura 3. A ventriculografia apresentava VE com volumes discretamente aumentados devido à hipocinesia difusa, fração de ejeção de 46% (A) e progressão importante da lesão do 1/3 proximal submetida à angioplastia em 1979 (B). O USIC mostrou uma área luminal de 3,22 mm^2^ (C). ACEsq apresentava ótima evolução angiográfica nos locais submetidos a implante de *stents* (D, E). Por apresentar quadro de AE e com a progressão da placa aterosclerótica do 1/3 proximal da ACD optou-se pelo implante de dois *stents* Taxus® 4,0*16 mm e 4,0*12 mm (F), guiados com USIC (G, H). Os dois *stents* prévios nos 1/3 médio e distal apresentavam hiperplasia neointimal de grau discreto pela avaliação com USIC (I, J). Na avaliação angiográfica e com USIC dos *stents* implantados previamente em TCESQ e ADA, observou-se uma ótima evolução com presença de discreto grau de hiperplasia neointimal (L-O).

Após 41 anos de pioneirismo, seguindo com entusiasmo a técnica que Grüntzig nos ensinou, oferecemos aos nossos pacientes o que há de mais novo dentro da cardiologia intervencionista, buscando sempre novas tecnológicas para fortalecer cada vez mais o tratamento da doença coronariana.

Ao acompanhar a trajetória do paciente A. S. O., tivemos a grandiosa oportunidade de conhecer os aspectos morfológicos desta patologia coronária, que é evolutiva e não tem cura. Foi possível acompanhar todo o avanço terapêutico e o diagnóstico por imagem (angiografia, USIC e OCT), desde a utilização do primeiro cateter-balão até a primeira geração de *stents* farmacológicos.

Após 34 anos de evolução, o paciente A. S. O. faleceu em 2013 em virtude de causas neurológicas. Aos seus familiares e em sua memória fica nosso eterno agradecimento pela confiança depositada em nossa equipe. Além disso, ressaltamos a importância dos colegas Dr. Flávio Nogueira (*in memoriam*) e Dr. Donaldo Pereira Garcia (*in memoriam*), que contribuíram sobremaneira para a evolução da técnica no Brasil.
