# Supplementary material for: A Evolução da Angioplastia Transluminal Coronariana na America Latina
Source: Arq Bras Cardiol. 2021 May 6;116(5):1007–10. [Article in Portuguese] doi: 10.36660/abc.20200927 (PMC8121465; doi:10.36660/abc.20200927)
Supplement: Translation

# Translation: The Evolution of Percutaneous Coronary Intervention in Latin America

**Authors:** Costantino Roberto Costantini, Rafael Michel de Macedo, Marcos Antônio Denk, Sergio Gustavo Tarbine, Lazaro Garcia, Mario Fernando Camargo Maranhão, Costantino Ortiz Costantini

**Affiliations:** 1 CuritibaPR Brazil Hospital Cardiológico Costantini Ltda, Curitiba, PR - Brazil; 2 CuritibaPR Brazil Santa Casa de Curitiba, Curitiba, PR - Brazil

**Keywords:** Angina Pectoris, Cineangiography/history, Angioplasty, Balloon, Coronary/history, Angioplasty Balloon, Coronary/trends, Stents, Myocardium/metabolism, Clinical Evolution

In August 1979, two years and one month after the first percutaneous coronary intervention (PCI) was performed in the world by Andreas Grüntzig,^1^ we received at Santa Casa de Misericordia in Curitiba (SCMC) a 55-year-old male patient (A.S.O.) with Canadian Cardiovascular Society (CCS) class 2 stable angina. Coronary angiography showed a severe (75-80 DS%) lesion in the middle segment of the Right Coronary Artery (RCA). The left coronary artery system did not have any significant atherosclerotic lesion and the left ventriculogram showed mild inferior wall hypokinesia with competent mitral and aortic valves.

That was the opportunity to put into practice the technique described by Gruntzig et al.^1^ for the first time in Latin America. The clinical presentation and the angiographic characteristics of the lesion met all the criteria described by Gruntzig: Single lesion, short length (<10mm), proximal arterial segment, without vasospasm, concentric, non-calcified and feasible for CABG. Thus, after discussing it with the clinical and cardiovascular surgery staff, it was decided to propose the dilation of the RCA obstruction to the patient as an attempt to treat his coronary disease.

After patient consent was obtained, on August 10, 1979 he was submitted to PCI as described by Costantini et al.^2^. After the PCI, the severe RCA lesion was reduced to a mild lesion (15-20 DS%). Despite the good angiographic result, there was concern about the heart muscle metabolism. Thus, in the absence of another method for myocardial ischemia assessment, a metabolic evaluation was performed by extracting a blood sample from the coronary sinus during temporary pacemaker-induced tachycardia pre- and post-PCI for evaluation of lactic acid levels. This evaluation confirmed the adequate oxygen supply to the heart muscle after PCI as demonstrated in Figure 1.^2^

**Figure 1:** A) Proximal RCA lesion before PCI, B) Proximal RCA lesion post-PCI, C) Coronary Sinus Lactate level assessment pre- and post-PCI.

During the subsequent years, the patient (A.S.O) was closely monitored regarding the coronary heart disease evolution. Other treatments were performed over time and new techniques and technologies were used. Figure 2 and Table 1 show patient evolution between 1982 and 2009.

**Figure 2:** Therapeutical evolution between 1982 and 2009 (A.S.O)

**Table 1:** Description of the patient's evolution and treatment (A.S.O.) between 1982 and 2009

Date	Exam	Diagnostics	Conduct
1982	Cardiac Catheterization 3 years of FU	LV: RCA preserved systolic function with maintained angiographic result, LMT: mild lesion, LAD and LCX: mild lesions;	Clinical Treatment
1993	Cardiac Catheterization 14 years of FU	LV: preserved systolic function, RCA: mild lesion in proximal LMT segment: mild lesion, LAD: moderate lesion in the proximal segment and severe lesion in the mid segment, LCX: severe lesion in the distal segment.	Rotational atherectomy PCI of LAD lesion and balloon PCI of LCX lesion
1997	Cardiac Catheterization 14 years of FU	LVEF: preserved systolic function, RCA: mild lesion in the proximal segment and severe lesion in the distal segment with ulcerated plaque on IVUS assessment, LMT: mild lesion, LAD: angiographic restenosis of rotational atherectomy PCI LCX: mild lesion in the distal segment;	PCI with 4.0x15 mm Palmaz-Schatz stent implantation in the distal RCA; Balloon PCI of LAD
1999	Cardiac Catheterization 20 years of FU Stable Angina	LV: preserved systolic function, RCA: mild lesion in the proximal segment; good angio and IVUS outcome of the distal stent, LMT: mild lesion, LAD: good angiographic outcome, LCX: good angiographic outcome;	Clinical treatment
2003	Cardiac Catheterization 24 years of FU Stable Angina	LV: preserved systolic function, RCA: mild lesion in the proximal segment, good angio and IVUS outcome of the distal stent, LMT: mild lesion, LAD: angiographic restenosis, ulcerated plaque in the LCX ostium: in stent 40% DS;	Proximal and mid LAD IVUS-guided DES PCI (CYPHER 2.75x18 & 2.75x33 mm). Ulcerated ostial plaque evaluated by IVUS and maintained in clinical treatment because it had preserved luminal area.
2005	Cardiac Catheterization 26 years of FU Stable Angina	LV: preserved systolic function, RCA: mild lesion in proximal segment; good angio and IVUS outcome of the distal BMS, LMT: moderate lesion LAD: severe ostial lesion, good angiographic and IVUS evolution of DES, LCX: angiographic restenosis;	Distal LCX (TAXUS stent) & ostial LAD (TAXUS stent) IVUS-Guided DES PCI
2007	Cardiac Catheterization 28 years of FU Stable Angina	LV: preserved systolic function, RCA: Moderate lesion in the proximal RCA and good angio and IVUS outcome of the distal BMS, LMT: severe lesion LAD: good angiographic and IVUS evolution of DES LCX: good angiographic and IVUS evolution of DES;	LMT IVUS-Guided DES PCI (TAXUS 4.0x28 mm)
2008	Check-up Multi-Slice Computed Tomography/ Cardiac Nuclear Scan Test	Normal perfusion, LV with preserved systolic function, RCA: moderate to severe lesion (60-70%) in the proximal segment and good evolution of the distal BMS;	Clinical Treatment
2009	Cardiac Catheterization 30 years of FU Stable Angina	RCA: Virtual Histology and OCT assessment of the proximal segment showing intermediate luminal area stenosis with a large Necrotic core and TCFA. Severe stenotic and ulcerated lesion in the mid segment.	Mid RCA IVUS-Guided BMS (3.5x18mm) PCI (3.5x18 mm). The proximal RCA lesion was not submitted to any intervention and maintained on clinical treatment.

RCA: right coronary artery; LMT: left main trunk; LCX: left circumflex artery; LAD: left anterior descending artery; PCI: percutaneous coronary intervention; IVUS: intravascular ultrasound; OCT: optical coherence tomography; BMS: bare metal stent; DES: drug-eluting stent; TCFA: thin-cap fibroatheroma.

In March 2010, the patient returned with CCS 2 stable angina, being submitted to the 9^th^ cardiac catheterization, of which images are shown in Figure 03. The LV gram showed slightly increased LV volumes due to diffuse hypokinesia and a 46% ejection fraction (A) and significant progression of the proximal 1/3 lesion submitted to angioplasty in 1979 (B). The IVUS showed a luminal area of 3.22 mm^2^ (C). The LMT, LAD and LCX presented with an excellent angiographic evolution in sites submitted to stent implantation (D,E). Because of angina limitations and the progression of the atherosclerotic plate in the proximal RCA, it was opted to perform the IVUS-Guided DES PCI with two Taxus stents (4.0x16 mm and 4.0x12 mm) (F, G,H). The two previous stents in the middle and distal segments of the RCA showed mild neointimal hyperplasia in the IVUS assessment (I,J). In the angiographic and IVUS evaluation of previously implanted stents in the LMT and LAD, an optimal evolution was observed with the presence of a mild degree of neointimal hyperplasia (L-O).

**Figure 3:** A) LV; B) pre-intervention RCA; C) RCA IVUS assessment; D) LMT/LCX angiography; E) LAD angiography; F) post-intervention RCA; G,H) post-intervention RCA IVUS; I,J) RCA IVUS assessment of the mid and distal segments in previous stents; K-O) LMT/LAD angiographic and IVUS previous stents.

Forty-one years after a pioneering intervention, enthusiastically following the technique that Grüntzig taught us, we can offer our patients the newest technology in interventional cardiology, always seeking new methods to improve the treatment of coronary heart disease.

By following the trajectory of patient A.S.O., we had the great opportunity to learn about the morphological aspects of this coronary pathology, which is progressive and has no cure. It was possible to monitor all therapeutic and diagnostic imaging advances (angiography, IVUS and OCT) starting with the use of the first balloon-catheter to the first generation of drug-eluting stents.

After 34 years of evolution of the first angioplasty performed in Latin America, patient A.S.O. died in 2013 from neurological causes. To his family and in his memory, our eternal gratitude for the trust placed in our team.
